# Supplementary material for: Atomic structures of Coxsackievirus A6 and its complex with a neutralizing antibody
Source: Nat Commun. 2017 Sep 11;8:505. doi: 10.1038/s41467-017-00477-9 (PMC5593947; doi:10.1038/s41467-017-00477-9)
Supplement: Supplementary file 1 — Supplementary Information [file 41467_2017_477_MOESM1_ESM.pdf]

File name: Supplementary Information
Description: Supplementary figures, supplementary tables and supplementary references.

File name: Supplementary Movie 1
Description: The cryoEM density map and atomic model of CVA6 A-particle. First, the iso-contoured (radially colored) cryoEM density map, (viewed along 2-fold axis) self-rotates along north-south axis showing its 2f-channels, q3f-channels and other capsid surface features. Second, the cryoEM density of a protomer is segmented out and fitted with the atomic models of capsid protein VP1 (blue), VP2 (green) and VP3 (red). Then, the close-up views of residues side chains to exhibit the quality of the cryoEM map. Finally, four surface loops of VP1 are labeled.

File name: Supplementary Movie 2
Description: The cryoEM density map and atomic model of CVA6 A-particle complexed with Fab-1D5. The iso-contoured (radially colored) cryoEM density map of CVA6 A-particle-1D5 immune complex (viewed along 2-fold axis) self-rotates along the north-south axis to demonstrate that five Fab-1D5 bind at each 5-fold vertex. Then, atomic model of 3 capsid proteins and variable domains of Fab-1D5 are fitting into the asymmetric unit of the map. Finally, the atomic model of entire capsid is created and displayed.

File name: Supplementary Movie 3
Description: The interaction interface between the CVA6 A-particle and the Fab-1D5. A segmented density (grey) including an asymmetric unit of CVA6 A-particle and the variable domain (light chain, orange; heavy chain, pink) of the Fab-1D5 is fitted with their atomic models. Then, the view is zoomed in to show the details of the A-particle-Fab interaction interface, where the side chains of critical residues are displayed and surface loops of VP1 are labeled.

File name: Peer review file
Description:

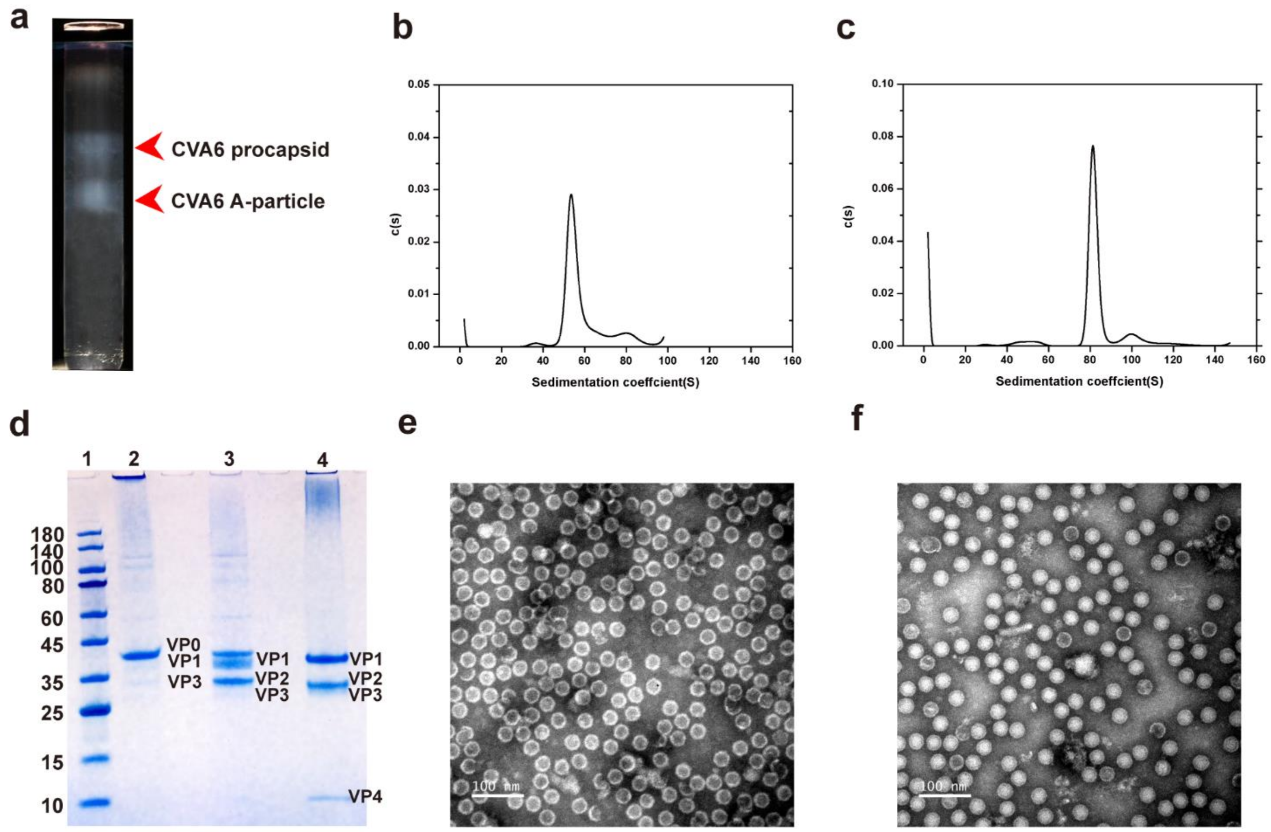

Supplementary Figure 1. CVA6 virus purification and characterization

(**a**) Two bands observed after zonal ultracentrifugation on a 15 to 45% (w/v) sucrose density gradient at 112,074 g for 3.5 h. With the absorbance ratio (λ260/λ280) of 0.79 and 1.65 respectively, the top band contains empty particles and the bottom band contains full particles (with RNA genome). **(b, c)** Sedimentation analysis of two types of particles. The particles in two bands of **(a)** exhibit major peak values of sedimentation coefficients s(T,b) of 54S and 81S respectively, where T is 4 °C and b 0.2 M PBS, equivalent to 84S and 128S at 20 °C[1]. We will thereafter refer to the particles in the top band as procapsid and those in the bottom band as A-particle. **(d)** Protein compositional analysis of the two types of CVA6 particles by SDS-PAGE. Lane 1, molecular mass markers; lane 2, procapsids; lane 3, A-particles and lane 4, CVA16 full particles (the control). The procapsids contain VP0, VP1 and VP3, whereas the A-particles contain VP1, VP2 and VP3, but no VP4. In contrast the CVA16 full particles contain all four structural proteins VP1, VP2, VP3 and VP4. **(e, f)** Negative stain electron microscopy of CVA6 procapsids **(e)** and A-particles **(f)**.

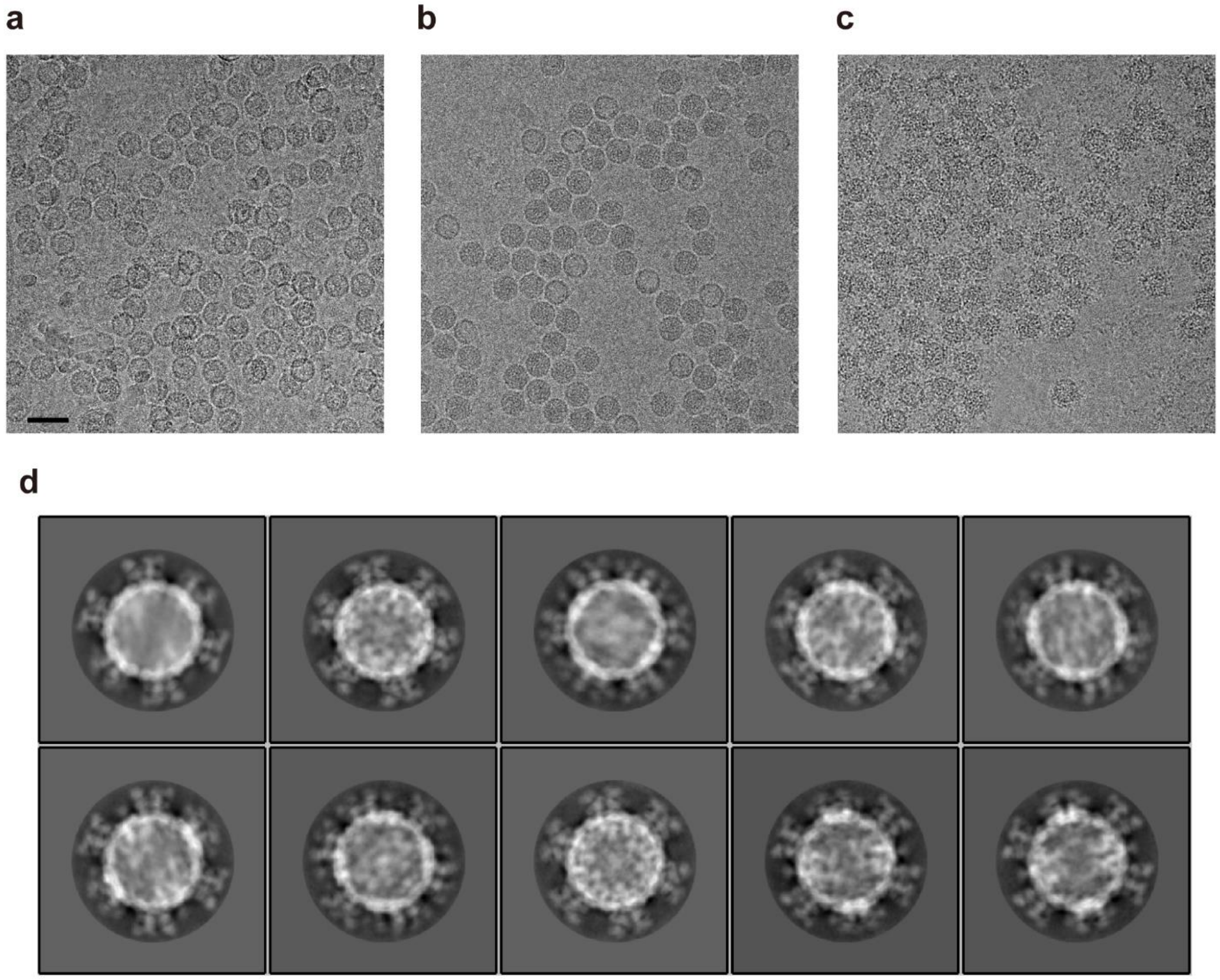

Supplementary Figure 2. CryoEM micrographs and image processing of CVA6 procapsid, A-particle and CVA6 A-particle-1D5 complex.

(a-c) Aligned (from 7 movie frames) micrographs of CVA6 procapsid **(a)**, A-particle **(b)** and CVA6 A-particle-1D5 complex **(c)**, scale bar = 50 nm. **(d)** Representative 2D class averages of CVA6 A-particle-1D5 complex particle images. A total of 34 well-aligned classes (12067 particles) were selected for subsequent 3D classification, and the first 10 mostly populated classes (6386 particles) were shown in **(d)**. Fab densities are clearly visible surrounding the outside of the virus capsid shell. White represents densities of proteins or genome in **(d)**.

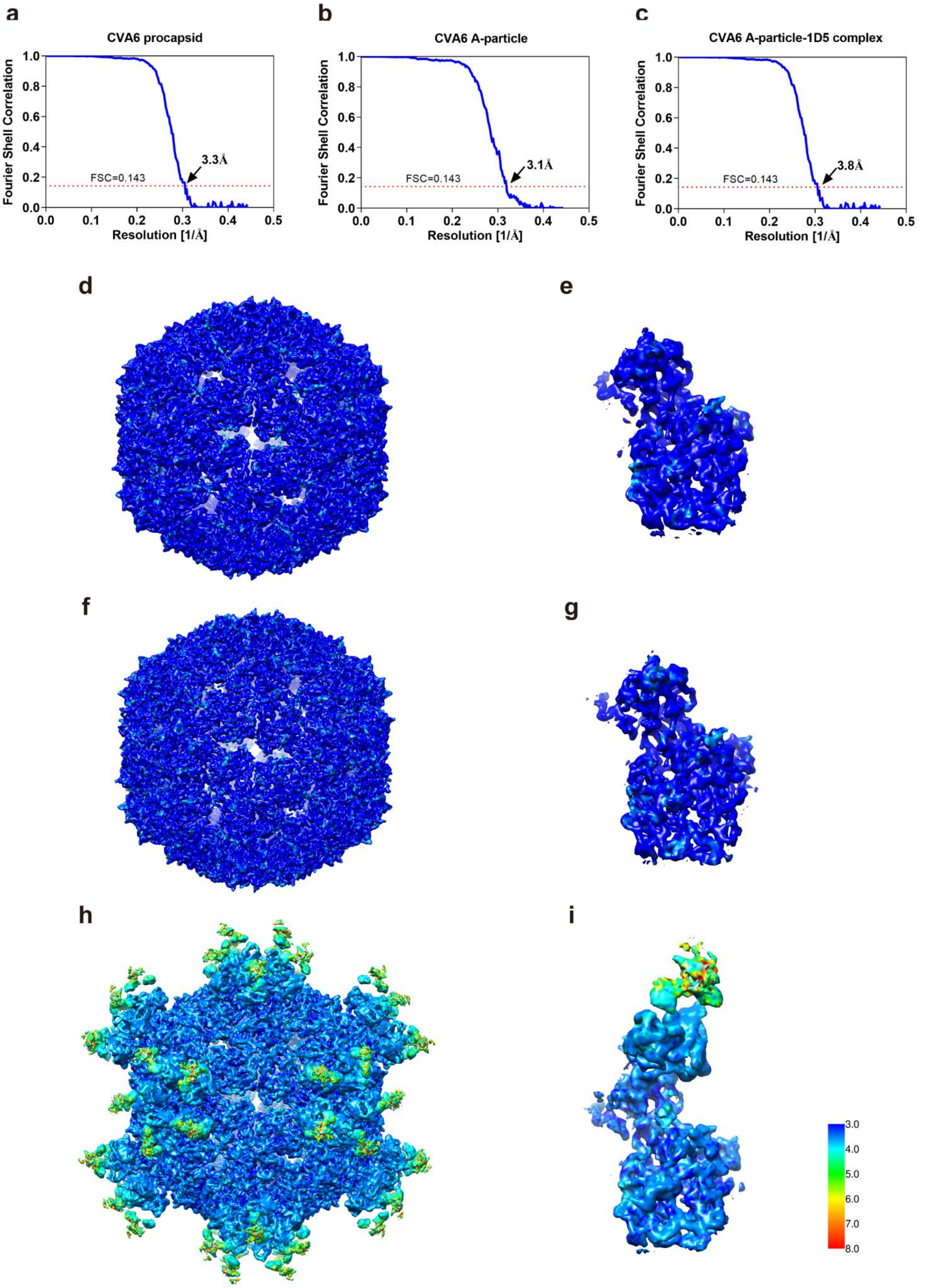

a
CVA6 procapsid
Fourier Shell Correlation
Resolution [1/Å]
FSC=0.143
3.3Å
b
CVA6 A-particle
3.1Å
c
CVA6 A-particle-1D5 complex
3.8Å
d
e
f
g
h
i
3.0
4.0
5.0
6.0
7.0
8.0

Supplementary Figure 3. Global and local resolution analysis.

(a, d, g) The gold standard FSC curve of the final density maps of CVA6 procapsid **(a)**, A-particle **(d)** and CVA6 A-particle-1D5 complex **(g)**. **(b, e, h)** Maps showing the local resolution (estimated by Resmap) variations in the CVA6 procapsid **(b)**, A-particle **(e)** and CVA6 A-particle-1D5 complex **(h)**. The resolution of the Fab densities distributed from ~3.8 Å resolution at interaction interface in variable domains to ~6.5 Å at distal ends of constant domains. **(c, f, i)** The details of local resolutions in asymmetric units of CVA6 procapsid **(c)**, A-particle **(f)** and CVA6 A-particle-1D5 complex **(i)**.

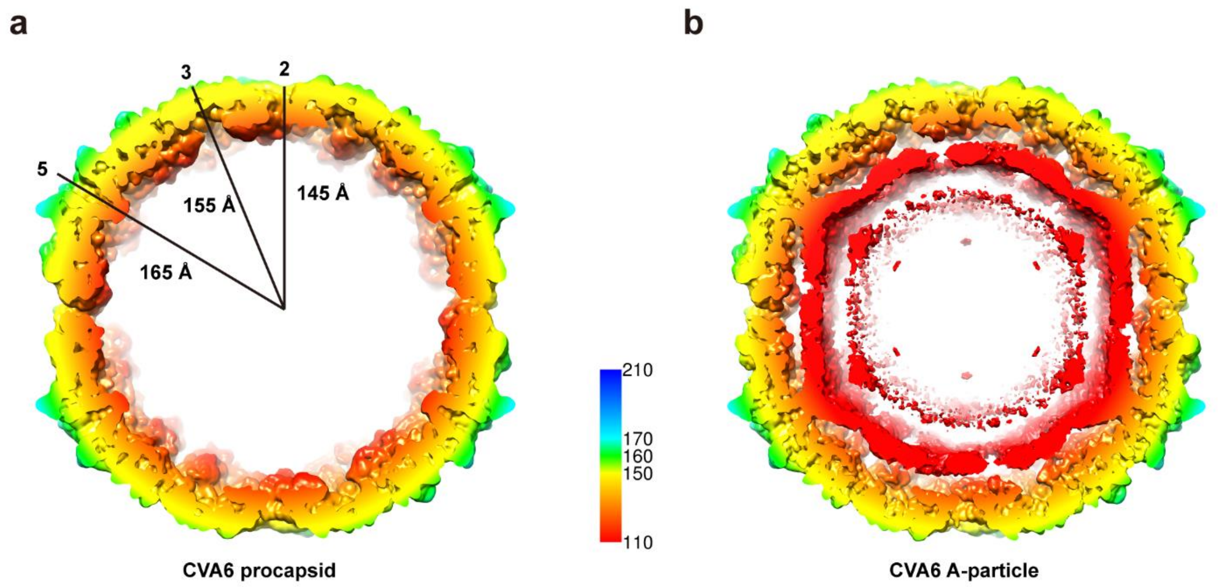

Supplementary Figure 4. The central sections of the cryoEM map (radially colored, displayed at 1.5σ) of the CVA6 procapsid **(a)** and A-particle **(b)**. The map of the procapsid shows no genome density whereas the CVA6 A-particle map does exhibit genome density (red) inside the capsid shell. Both particles have radii of 145, 155 and 165 Å along 2-,3- and 5-fold axes, and the lengths of the measurements are indicated with black lines along the axes in **(a)**.

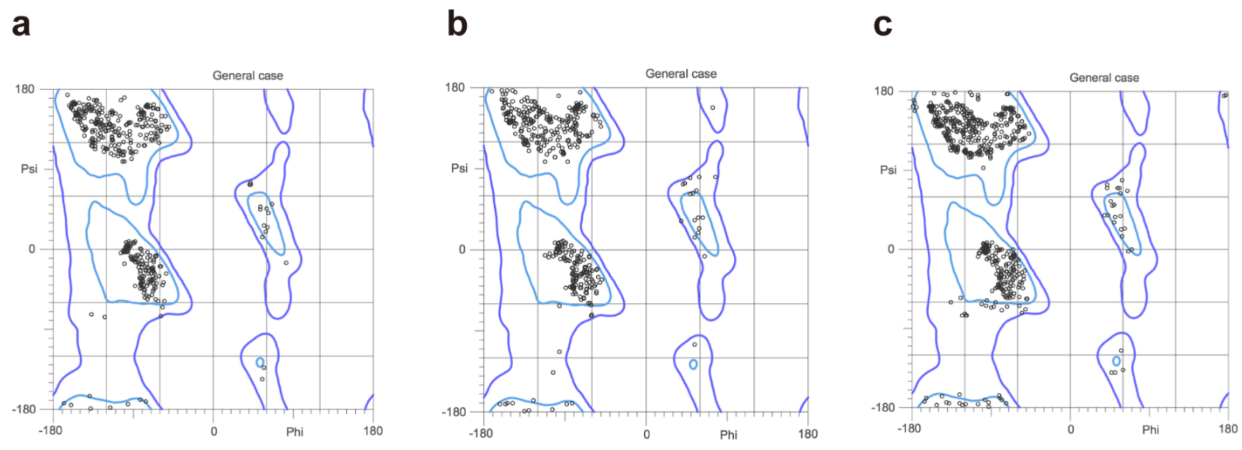

Supplementary Figure 5. Ramachandran plots of the atomic models of CVA6 procapsid **(a)**, CVA6 A-particle **(b)**, and CVA6 A-particle-1D5 **(c)**, respectively.

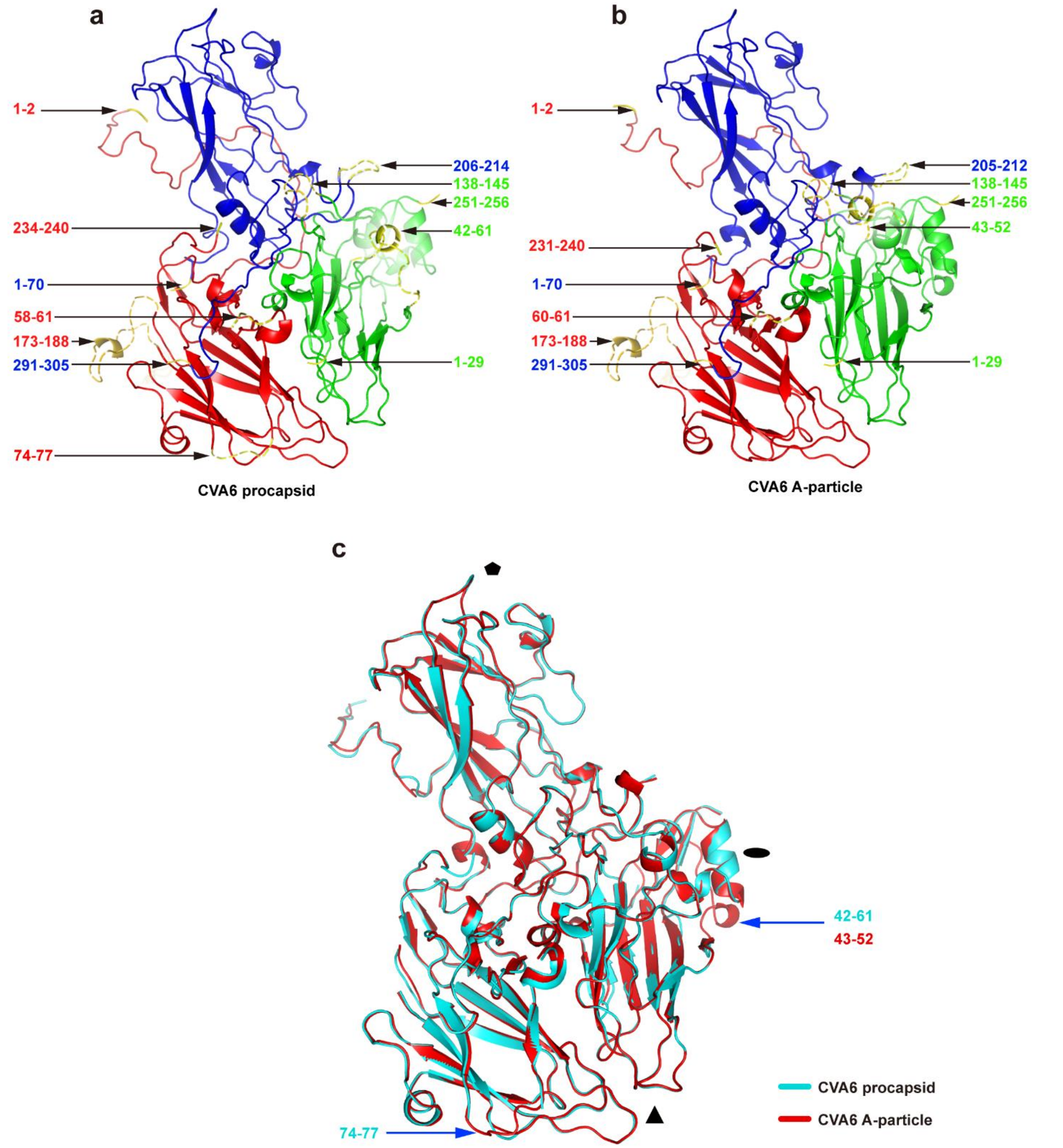

Supplementary Figure 6. Missing residues in the atomic models of procapsid and A-particle. The atomic models of procapsid **(a)** and A-particle **(b)** protomers plus the superimposition **(c)** demonstrate their essentially identical structures yet with two regions (blue arrows), where A-particle contains more identifiable residues than procapsid.

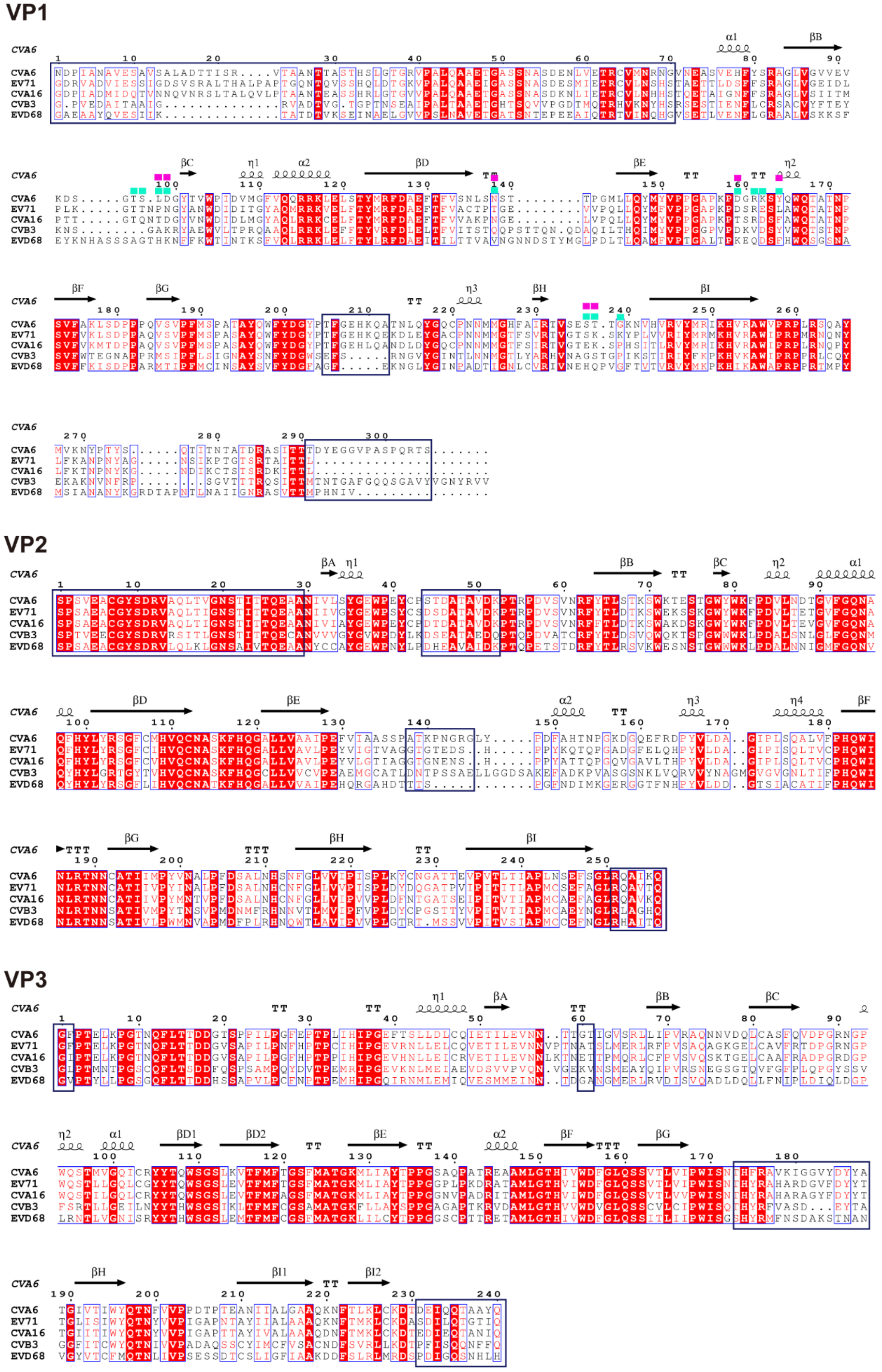

Supplementary Figure 7. Espript representation of a structure-based sequence alignment of VP1, VP2

and VP3 of the CVA6 (GenBank accession no. KR706309) with four other representative picornaviruses EV71, CVA16, CVB3 and EVD68 (GenBank accession no. FJ600325, FJ198212, M88483, AY426531 respectively). Cyan squares mark the critical residues in neutralizing antigenic epitopes against 1D5, magenta squares identify these residues involved in forming hydrogen bonds with Fab. Dark blue boxes highlight those unidentifiable residues in the CVA6 A-particle cryoEM map. Residue numbering is based on that for CVA6.

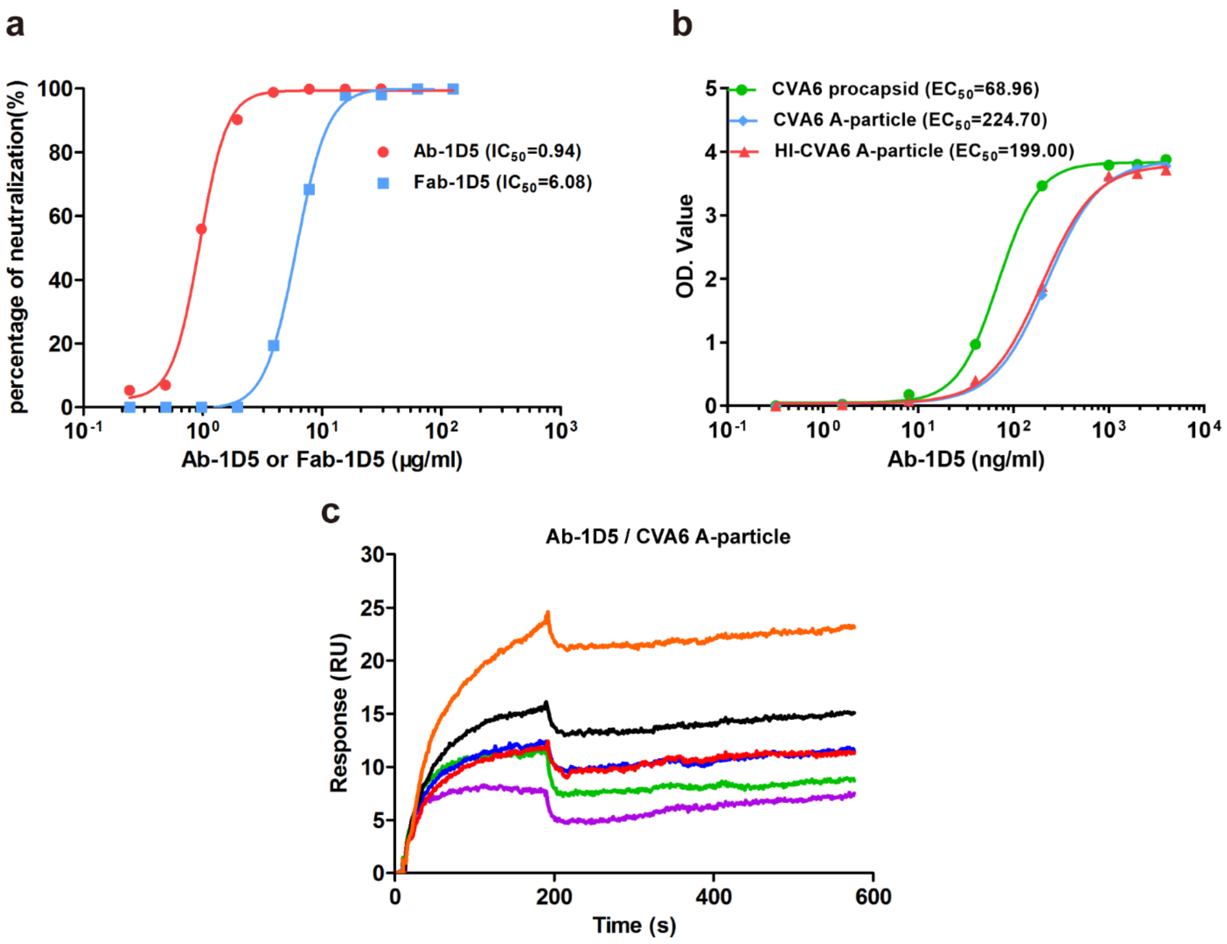

Construct	$K_a(M^{-1}\cdot s^{-1})$	$K_d(s^{-1})$	$K_A(M^{-1})$	K_D(nM)
Ab-1D5	1.53×10^5	6.34×10^{-4}	2.41×10^8	4.14

Supplementary Figure 8. Neutralizing potencies and binding affinities of Ab-1D5 against CVA6. **(a)** The neutralizing potency of Ab-1D5 and Fab-1D5 evaluated at different concentrations with neutralization assay. The IC_{50} of Ab-1D5 was determined to be 0.9 μg/mL, while that of Fab-1D5 was 6.1 μg/mL, both elicit a high neutralizing titer against CVA6. **(b)** Binding affinities of Ab-1D5 to CVA6 particles (procapsid, A-particle, and HI-A-particle) evaluated with indirect ELISA. The EC_{50} was calculated with non-linear regression fitting curves. The avidity of Ab-1D5 for CVA6 A-particle (EC_{50} = 201.4 ng/mL) was slightly better than that for HI-A-particle (EC_{50} = 187.3 ng/mL). **(c)** The affinity constants of Ab-1D5 with CVA6 A-particles quantified by SPR in Biacore 3000. Binding curves obtained by passing different concentrations of A-particles over biotinylated Ab-1D5 immobilized on a biosensor surface are shown in the top panel. The kinetic values showing in the bottom panel were obtained by simultaneously fitting the association and dissociation responses to a 1:1 Langmuir binding model (K_D, kinetic). The data are representative of two independent experiments. Ab-1D5 exhibits a high binding

affinity for CVA6 A-particles as revealed by an equilibrium dissociation constant (K_D) value of less than 10 nM.

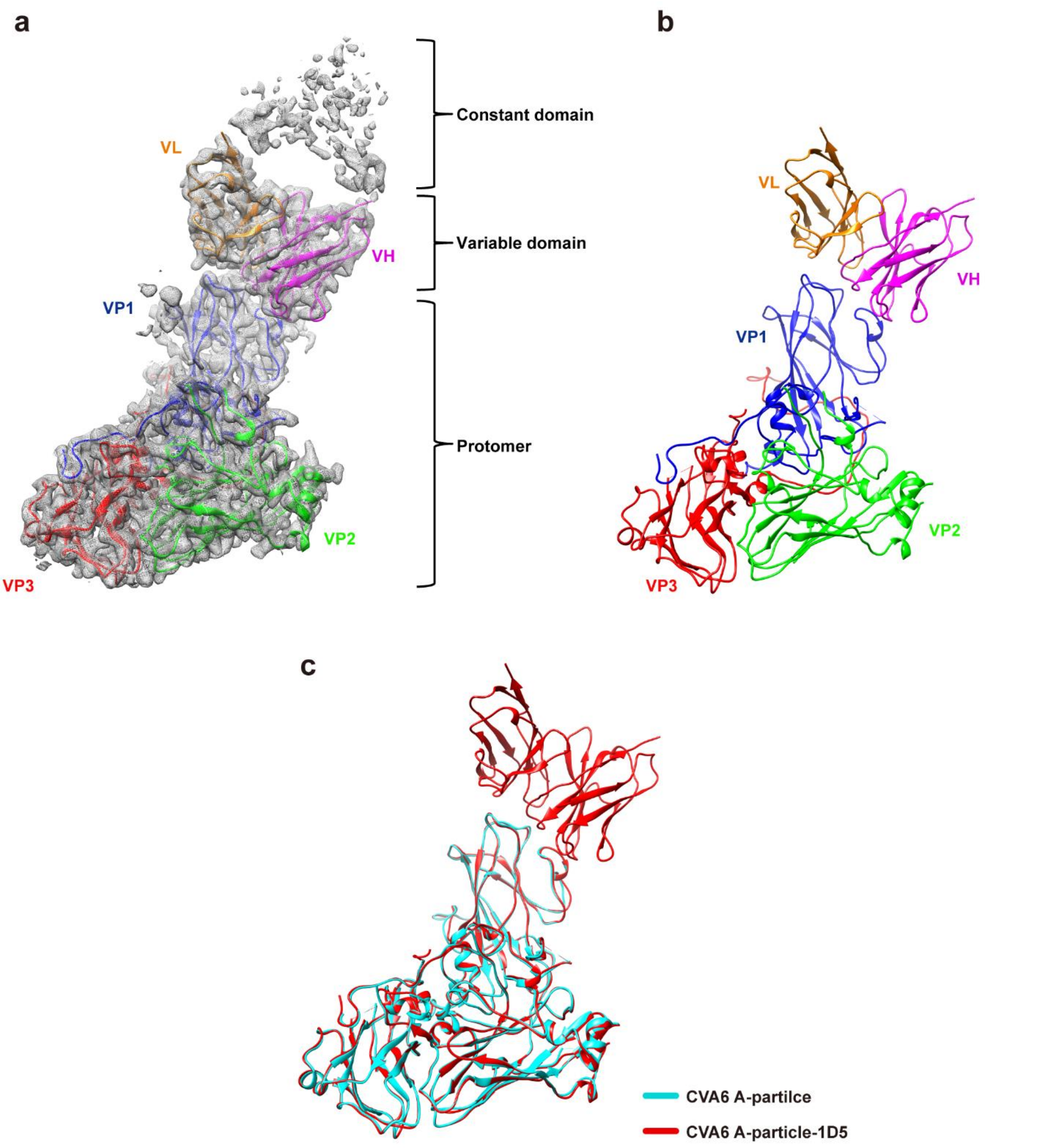

Supplementary Figure 9. (a) The segmented asymmetric unit of the CVA6-A-particle-1D5 immune-complex density map (gray) fitted with atomic models (ribbon diagrams) of three capsid proteins and the variable domain (light chain, VL, orange; heavy chain, VH, magenta) of the Fab-1D5. The constant domain of the Fab exhibits very weak densities at this contour level (4σ). **(b)** The atomic models of three capsid proteins and variable domain of the Fab-1D5. **(c)** The superposition of the protomers in the CVA6-A-particle-1D5 complex and in A-particle demonstrate essentially identical structures of protomers with or without 1D5 binding.

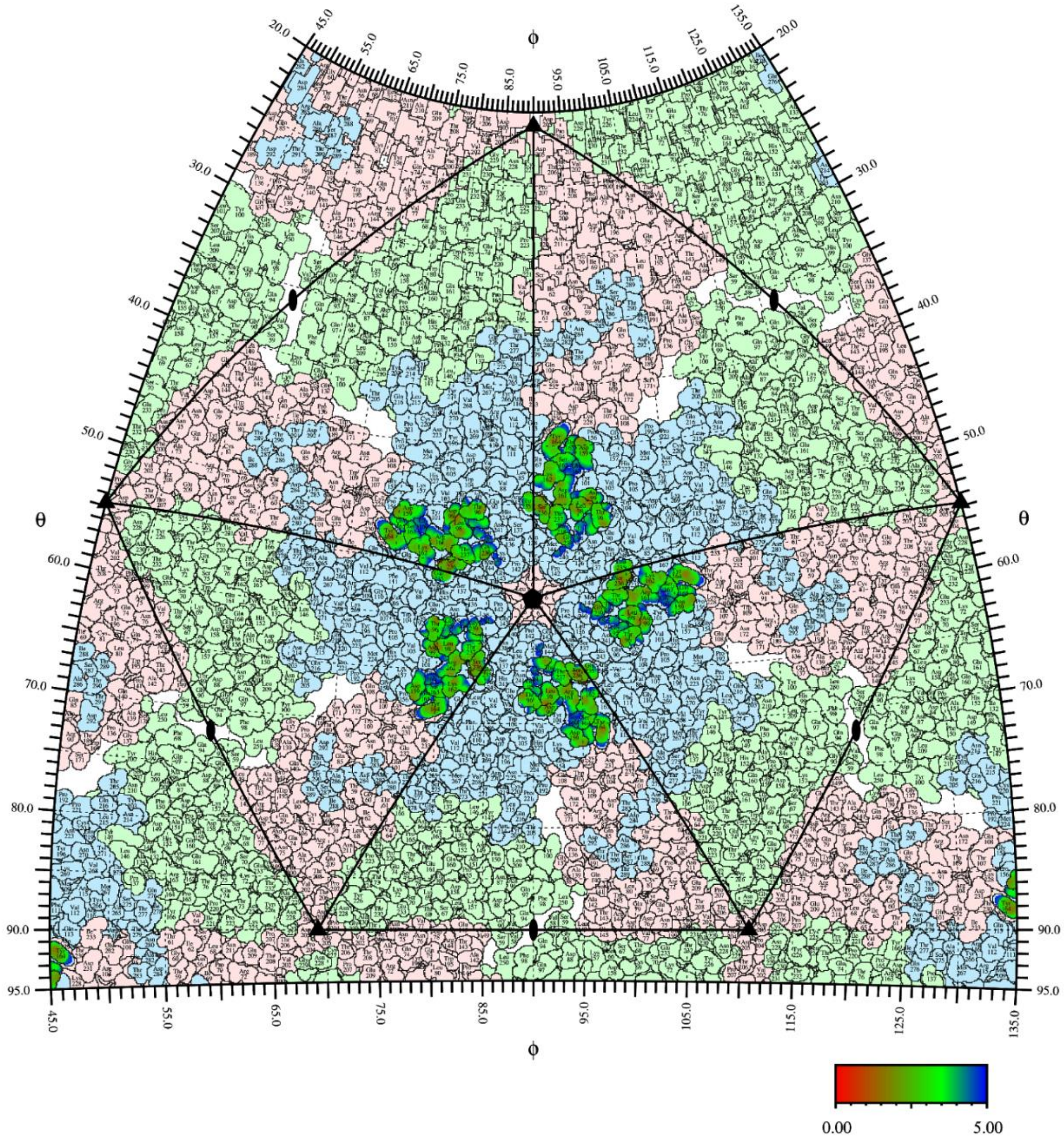

Supplementary Figure 10. Roadmap of five Fab-1D5 footprints on the CVA6 A-particle capsid at a 5-fold vertex. The surface of the CVA6 A-particle is shown as a stereographic projection. The polar angles θ and ϕ indicate latitude and longitude, respectively. The Fab-1D5 footprints are colored according to the atom distance of particle surface to Fab. The border of one asymmetric unit is outlined with black lines, and the 2-, 3- and 5-fold icosahedral symmetry axes are labeled with black ellipses, triangles, and pentagons, respectively. The amino-acid residues of CVA6 A-particle are also shown in the expanded view. The surfaces of capsid protein VP1, VP2 and VP3 are colored in light blue, green and pink, respectively.

Supplementary Table 1. Regions with unidentifiable residues in atomic models.

Capsid protein	Residues (a.a.)	Regions missing density	
		CVA6 procapsid	CVA6 A-particle
VP0(VP4+VP2)	325	1-69 (VP4); 1-29 (VP2); 42-61 (VP2); 138-145 (VP2); 251-256 (VP2)	/
VP1	305	1-70; 206-214; 291-305	1-70; 205-212; 291-305
VP2	256	/	1-29; 43-52; 138-145; 251-256
VP3	240	1-2; 58-61; 74-77; 173-188; 234-240	1-2; 60-61; 173-188; 231-240
VP4	69	/	/

Supplementary Table 2. Structural comparison (r.m.s.d.) of capsid proteins (upper) and VP1 surface loops (lower) of CVA6, CVA16 and EV71 in different particle forms.

Particles	CVA6 A-particle			
	VP1	VP2	VP3	Protomer
CVA6 procapsid	0.3 / 211*	0.4 / 193	0.5 / 203	0.4 / 607
CVA6 A-particle-1D5	0.4 / 213	0.4 / 203	0.4 / 210	0.4 / 626
CVA16 A-particle	1.0 / 212	0.8 / 201	0.6 / 208	0.8 / 621
EV71 A-particle	1.2 / 210	0.7 / 202	0.8 / 206	0.9 / 618
EV71 mature virion	1.8 / 202	0.6 / 203	1.3 / 206	1.3 / 611

* first number means r.m.s.d., second number means align atoms.

Particles	CVA6 A-particle				
	BC loop	DE loop	EF loop	HI loop	All 4 loops
CVA6 procapsid	0.3 / 10*	0.3 / 10	0.3 / 14	0.4 / 9	0.3 / 43
CVA16 A-particle	2.3 / 10	1.2 / 10	1.6 / 14	2.3 / 9	1.8 / 43
EV71 A-particle	2.0 / 8	1.7 / 10	1.4 / 14	2.6 / 9	1.9 / 41
EV71 mature virion	3.2 / 7	3.4 / 6	2.1 / 14	3.0 / 8	2.8 / 35

* first number means r.m.s.d., second number means align atoms.

Supplementary Table 3. CVA6 A-particle-1D5 contacts in atomic model of immune-complex.

Critical residues in epitope		Critical residues on Fab-1D5												
		CDRL1	CDRL2	FR3	CDRL3			CDRH2				CDRH3		
		ASN36	SER56	TYR66	HIS107	TYR108	THR109	GLY59	GLY62	GLY63	TYR64	LEU110	TYR112	TYR113
**BC loop**	THR96			√										
	SER97													√
	LEU98		√		√									**H**
	ASP99													**H**
**EF loop**	ASP159							√	**H**	**H**				
	ARG161											√	√	
	LYS162										√	√		
	TYR164									**H**				
**HI loop**	SER236					√	√						**H**	
	THR237					**H**							√	
	GLY239	√												
**DE loop***	ASN138					**H**								

Node: [√]interaction residue; [H]Hydrogen bond interaction; [*]adjacent VP1 DE loop

Supplementary References

1. Tanford, C. *Physical chemistry of macromolecules*, 710 p. (Wiley, New York,, 1961).
